# Discordant myocardial perfusion defects and coronary artery disease – a multimodality imaging journey in the natural history of cardiac sarcoidosis

**DOI:** 10.1007/s10554-024-03232-6

**Published:** 2024-09-13

**Authors:** Andrei Galafton, Ronny R. Buechel, Fran Mikulicic, Philipp A. Kaufmann, Andreas A. Giannopoulos

**Affiliations:** 1 Nuclear Medicine Department, Cardiac Imaging, University Hospital Zurich, Raemistrasse 100, Zurich, 8091 Switzerland; 2 Department of Cardiology, University Heart Center, University Hospital Zurich, Raemistrasse 100, Zurich, 8091 Switzerland

**Keywords:** Cardiac sarcoidosis, Myocardial perfusion defects, PET, Inflammation, Discordance, Microvascular disease

## Abstract

Cardiac sarcoidosis is a rare form of systemic sarcoidosis characterized by formation of non-caseating granulomas in the myocardium, leading to heterogeneous manifestations, including conduction disturbances, arrhythmias, and heart failure. Besides myocardial fibrosis and structural myocardial alterations, cardiac sarcoidosis can impact the epicardial and microvascular circulation, causing ischemia and regional microvascular dysfunction. We present a case of cardiac sarcoidosis with atypical initial presentation but with evidence of disease in myocardial perfusion imaging, which was overlooked since it did not correspond to bystander coronary artery disease. Multimodality imaging facilitated diagnosis and offered insights into the regional microcirculation impairment caused by sarcoidosis-induced inflammation.

## Introduction

Cardiac sarcoidosis (CS) is an inflammatory granulomatous cardiomyopathy of unclear etiology. Around 5% of the patients with systemic sarcoidosis have clinically manifest cardiac involvement, while 20–25% of patients might have clinically silent CS. [1] Prevalence of isolated CS is not well reported but is considered to affect up to 25% of patients with CS. [2, 3] The disease involves the recruitment and activation of macrophages or lymphocytes, leading to the formation of granulomas and subsequent myocardial fibrosis. Clinically, CS can manifest with conduction abnormalities, ventricular arrhythmias, and heart failure. The advent of advanced imaging techniques, such as cardiac MRI and ^18^F-FDG-PET/CT, has significantly enhanced the detection and management of CS, providing deeper insights into its pathophysiology. [4]

We present a case of a middle-aged man with atypical first presentation of cardiac sarcoidosis and imaging findings of disease involvement at myocardial perfusion imaging which were initially overlooked. Multimodality imaging enabled definite diagnosis and revealed the presence of ischemia and regional microvascular myocardial disease without concordance to bystander coronary artery disease (CAD). This case report aims to highlight the importance of considering inflammatory and infiltrative cardiomyopathies in the differential diagnosis when patients present with myocardial perfusion defects in the absence of concomitant coronary artery disease. It also demonstrates the interplay between myocardial inflammation and the regional impairment of coronary microcirculation.

## Case report

**Fig. 1:**
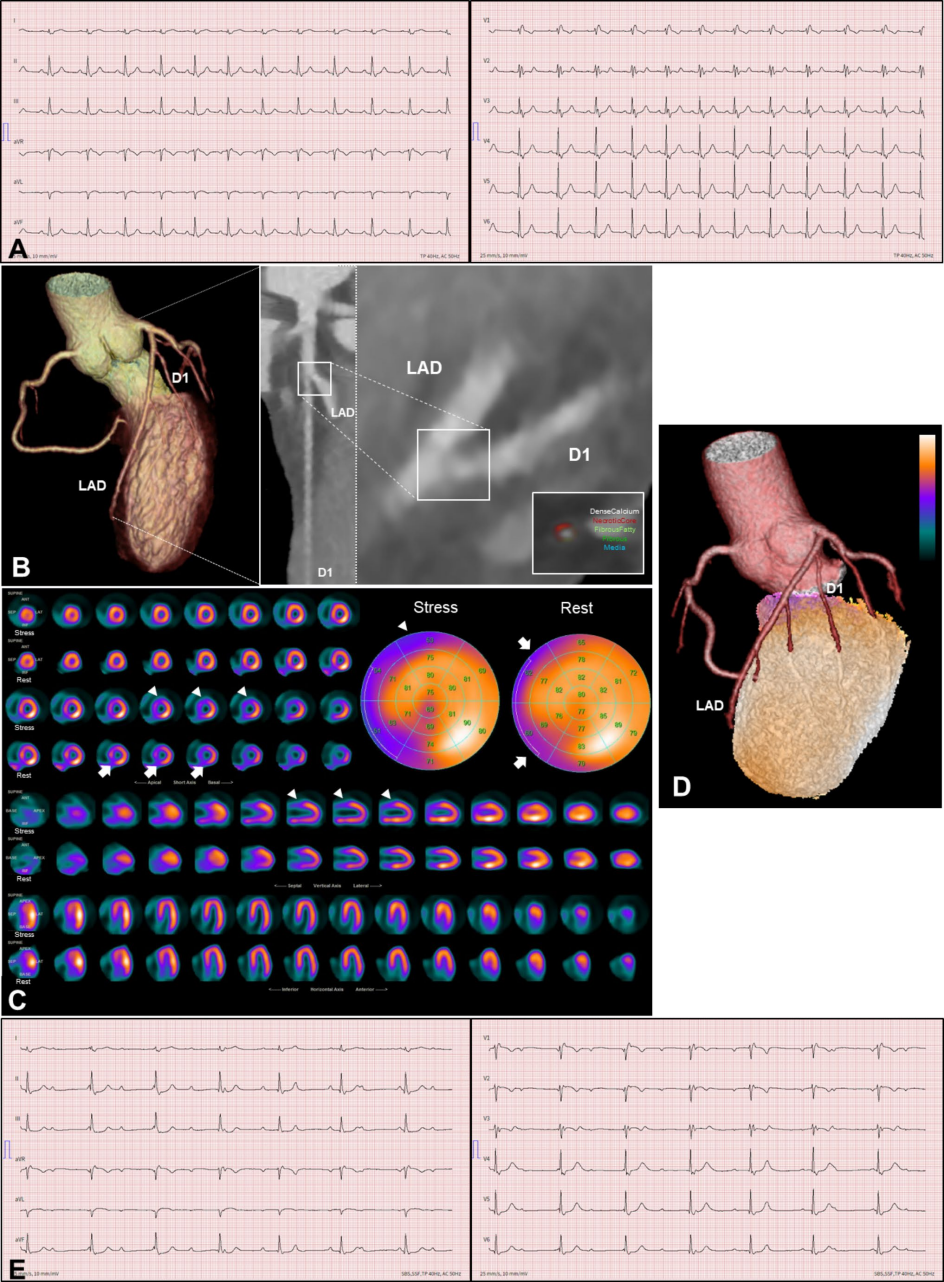
Initial investigation. ***A***: 12-lead ECG at first presentation, showing sinus rhythm and complete right bundle branch block. ***B***: CCTA demonstrating a non-stenotic, non-calcified plaque, encompassing necrotic core at the ostium of the D1. ***C***: ^99^mTc-SPECT-MPI with evidence of inferoseptal and anteroseptal fixed perfusion defect (arrows) and reversible perfusion defect anterobasal (arrowheads). ***D***: Fusion of CCTA with ^99^mTc-SPECT-MPI-Stress showing absence of perfusion defect in the D1 territory. ***E***: ECG at second presentation with first diagnosis of third-degree AV-Block

A previously healthy, athletic 50-year-old man presented with recent-onset intermittent and progressive dyspnea (NYHA class II) and exercise intolerance. Physical examination was unremarkable with vital signs within normal range. A 12-lead ECG showed sinus rhythm with complete right bundle branch block and normal repolarization (Fig. 1A). A TTE revealed normal LVEF (58%) without regional wall motion abnormalities. An acute coronary syndrome was excluded by serially slightly elevated high-sensitive troponin T but without dynamic changes (27 and 25 ng/l after 3 h; *n* < 14 ng/l) and normal creatine kinase. NT-proBNP and CRP were normal while LDL-Cholesterol was slightly elevated with 4.0 mmol/l (154 mg/dl). In order to exclude obstructive CAD and by low pre-test probability, a coronary CT angiography (CCTA) was performed, revealing absence of coronary calcifications (CAC score 0 AU) but an ostial, non-obstructive, low-attenuation plaque of the first diagonal branch (D1) (Fig. 1B). An ad-hoc, complementary, ^99m^Tc-SPECT myocardial perfusion (^99m^Tc-SPECT-MPI) scan demonstrated fixed inferoseptal and anteroseptal perfusion defects and a reversible anterior basal perfusion defect. Hybrid imaging with CCTA showed no correlation of the perfusion defects with the myocardium supplied by the D1 (Fig. 1C and D) and the MPI was reported as normal. Statin therapy was recommended and the patient scheduled for follow-up consultation. Two months later, the patient presented again with worsening dyspnea and extreme exercise intolerance. Although blood pressure was normal (127/66 mmHg), he was profoundly bradycardic (40 bpm) and ECG revealed a third-degree AV-Block (Fig. 1E). During rhythm monitoring alternating third-degree and 2:1 AV-Block was observed.

**Fig. 2:**
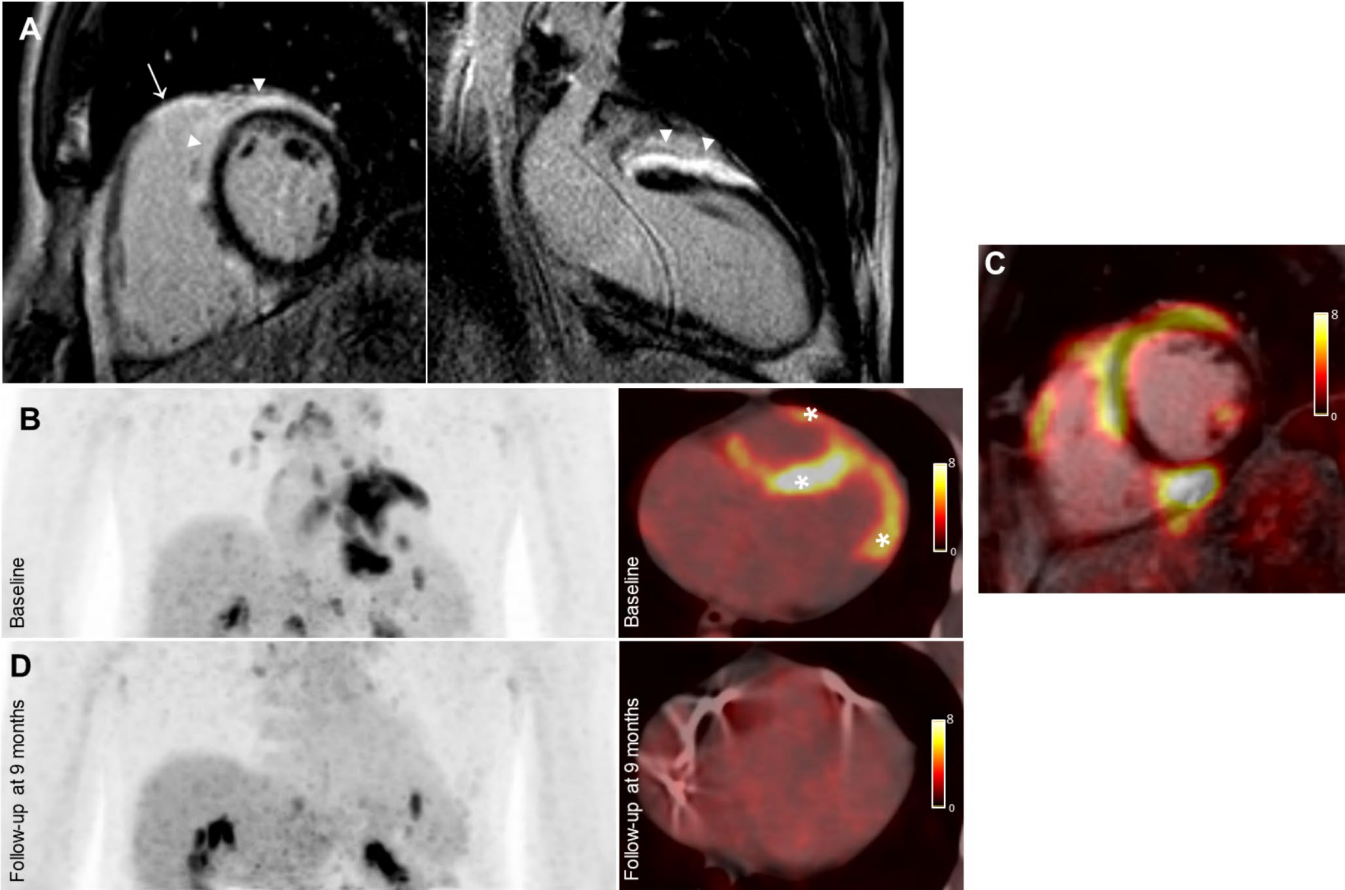
Advanced imaging. ***A***: Cardiac MRI short axis and 2-chamber views, showing epicardial late gadolinium enhancement of the LV (anterior, anteroseptal and inferoseptal – arrowheads) and the RV free wall (arrow). ***B***: Baseline ^18^F-FDG-PET/CT planar and axial images revealing multi-focal myocardial inflammation of the LV and the RV (asterisks). ***C***: Axial fused image of cardiac MRI and baseline ^18^F-FDG-PET/CT, depicting matching of the epicardial fibrosis with the myocardial inflammation. ***D***: Follow-up ^18^F-FDG-PET/CT, showing complete remission of the cardiac inflammation

As next diagnostic test, a cardiac MRI was performed demonstrating normal dimensions and function of the LV and RV without regional wall motion abnormalities, nevertheless revealing multifocal epicardial late gadolinium enhancement and elevated global T_1_- and T_2_-mapping values (Fig. 2A). To further investigate the suspicion of sarcoidosis with cardiac involvement, the patient underwent a cardiac ^18^F-FDG-PET/CT (with dietary preparation with a high-fat/low-carbohydrate diet for ≥ 24 h, fasting for at least 12 h, as well as intravenous administration of unfractioned heparin) showing, congruent to the fibrosis areas, myocardial inflammation of the LV myocardium and the RV wall (Fig. 2B-C). Furthermore, several, enlarged metabolic active mediastinal and hepatic portal lymph-nodes were noted. Following multidisciplinary discussion, the patient underwent bronchoscopy-guided, fine-needle aspiration biopsy of the mediastinal lymph-nodes, with histopathology confirming the presence of sarcoid-typical, non-necrotizing granulomas.

Corticosteroid and immunosuppressive therapy was initiated and following shared decision making with the patient, a primary prevention two-chamber defibrillator was implanted, accounting for the potentially pro-arrhythmogenic extend of LV-myocardial-fibrosis. A follow-up ^18^F-FDG-PET/CT at four months demonstrated regression of the cardiac inflammation activity and extent. Seven months following diagnosis, the patient suffered an electrical storm, successfully terminated with antitachycardia pacing and oral amiodarone was started. The pharmacological sarcoidosis management consisted mainly of intermittent high-dose corticosteroid and additional immunomodulatory therapy (initially with azathioprine and later adalimumab). At nine months follow-up, ^18^F-FDG-PET/CT revealed complete remission of the cardiac inflammation (Fig. 2D). No further cardiac MRI was performed due to anticipation of image susceptibility artifacts. The patient remained asymptomatic with no further ventricular arrhythmias, amiodarone was discontinued and low-dose bisoprolol was initiated.

**Fig. 3:**
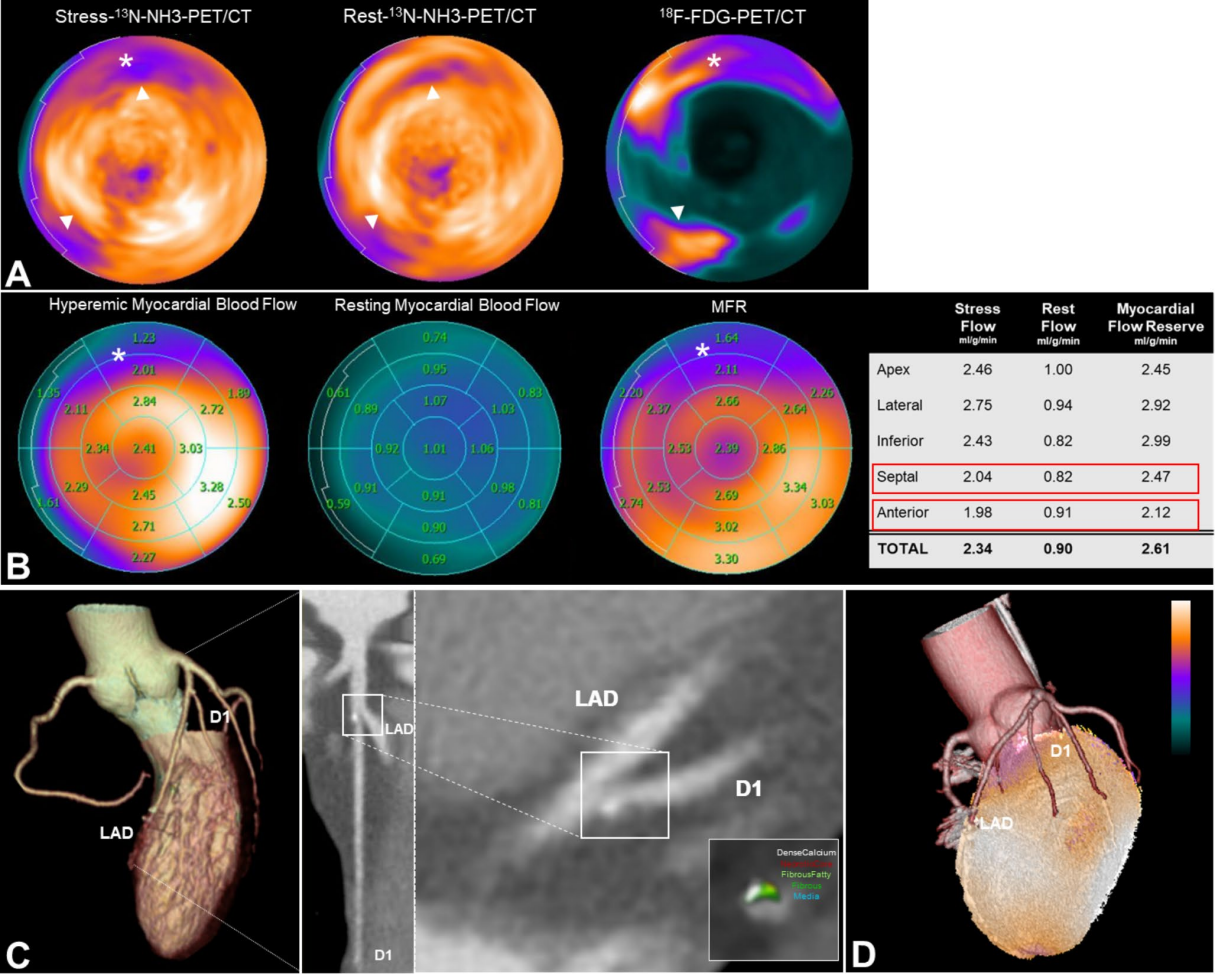
Follow-up Imaging. ***A***: ^13^N-NH_3_-PET/CT stress/rest polar plots demonstrating an inferoseptal (fibrosis/scar; arrowhead) and a smaller anterior-basal fixed perfusion defect along with an anterior/anteriorseptal-basal reversible perfusion defect (ischemia; asterisk); and baseline ^18^F-FDG-PET/CT polar plot demonstrating the matched, initially inflamed myocardial regions. ***B***: ^13^N-NH_3_-PET/CT myocardial blood flow analysis demonstrating regional (anterior/anteriorseptal) hyperemic myocardial blood flow and MFR reduction, congruent to the ischemic areas (asterisk). ***C***: Follow-up CCTA demonstrating discrete progression of the now fibrocalcific ostial D1 lesion, causing borderline obstruction (ca. 50% luminal area stenosis). ***D***: Fusion of follow-up CCTA with ^13^N-NH_3_-PET/CT, depicting mismatch of the abnormally perfused areas with the D1 territory

Three years after diagnosis, on routine consultation, ECG changes suggestive of ischemia were noted at cycle exercise test, triggering a non-invasive ischemia test with ^13^N-NH_3_-PET/CT. The latter revealed an inferoseptal and a smaller anterior fixed perfusion defect (fibrosis/scar) along with an anterior/anteroseptal ischemia (Fig. 3A). Regional myocardial blood flow reserve (MBFR) and hyperemic myocardial blood flow (MBF) were reduced, congruent to the abnormally perfused areas, denoting possible regional microvascular dysfunction (Fig. 3B). Based on the minimal coronary calcifications at ^13^N-NH_3_-PET/CT (CAC score 5 AU), a CCTA was repeated, showing solely local progression of the ostial D1 lesion, now causing borderline obstruction (ca. 50% luminal area stenosis) (Fig. 3C). Fusion imaging demonstrated that the atherosclerotic lesion did not cause any ischemia, with the perfusion defects matching rather the fibrosis and inflammatory areas of the LV-myocardium (Fig. 3D). Statin therapy was intensified and uptitration of beta-blocker was recommended.

## Discussion

Assessment of the presence and extend of myocardial fibrosis/scaring represents a key aspect in the diagnostic approach of patients with suspected CS [5]. CMR has been suggested as a useful initial diagnostic test, accounting for the excellent ability to detect fibrosis and its prognostic value. Similarly, assessment of myocardial perfusion with nuclear cardiology modalities, allows for identification of localized jeopardized (ischemic and fibrotic) myocardium, which might precede echocardiographic wall motion abnormalities. Perfusion defects might be attributed to the CS itself, provided that bystander CAD has been excluded. This was illustrated in the present case, whereby hybrid anatomical and functional imaging could acquit the atherosclerotic lesion, since perfusion-defect areas were supplied by normal coronary arteries.

Myocardial blood flow quantification with PET/CT enables assessment of global or, more frequently in the case of early stage CS, regional microvascular dysfunction. Sarcoid-mediated inflammation has been shown to be associated with regional impairment of epicardial and microvascular coronary function [6]. While the mechanism is unclear and certainly multifactorial, hyperemic MBF and MBFR have been reported to be lower in inflamed myocardium compared to non-inflamed areas in CS patients [7]. Inflammation has been also associated with coronary vasoconstrictive reactivity at the epicardial and microvascular level. Our patient had evidence of ischemia and regional coronary microvascular disease, not related to CAD, but co-localised with the initially inflamed anterior basal myocardium. This highlights the need for a more nuanced approach when assessing coronary endothelial and microvascular function, in contrast to the prevailing assumption of “global” coronary vasodilatory capacity through the functional interrogation of a single coronary artery during invasive physiology assessment. Although under immunotherapy the inflammation was metabolically in remission, there was still evidence of ongoing pathophysiological processes, which potentially cannot be detected by currently available imaging modalities. No recommendation exists regarding anti-ischemic therapy in CS, and optimization of the disease-specific therapy remains the mainstay.

In conclusion, discordant myocardial perfusion defects and coronary anatomy should be carefully interpreted within clinical context. Absence of coronary artery disease in arteries supplying abnormally perfused myocardium might, in certain cases, deserve a deeper diagnostic work-up, since inflammatory or infiltrative cardiomyopathies might present similarly. The natural history of CS and the pathophysiological mechanisms and effects of the current therapeutic approach can pose a clinical challenge. Multimodality imaging can provide insights into the structural, metabolic and coronary microcirculation level, thereby enhancing our understanding of the disease.

## Data Availability

No datasets were generated or analysed during the current study.
